# Evaluation of cystoprostatectomy on patients with prostate cancer extending to bladder: a retrospective study from single center

**DOI:** 10.1186/s12894-022-01068-7

**Published:** 2022-07-28

**Authors:** Xiaoliang Sun, Min Liu, Yong Zhao, Kang Leng, Haiyang Zhang

**Affiliations:** 1 Department of Urology, Shandong Provincial Hospital Affiliated to Shandong First Medical University, Jinan, 250021 Shandong China; 2 Department of Oncologic Chemotherapy, Shandong Second Provincial General Hospital, Jinan, 250022 Shandong China; 3 Knuppe Molecular Urology Laboratory, Department of Urology, School of Medicine, University of California, San Francisco, CA 94143 USA

**Keywords:** Cystoprostatectomy, Prostate cancer, Prostate cancer-specific survival, Quality of life

## Abstract

**Background:**

This is an exploratory research of cystoprostatectomy (CP) in treating prostate cancer (PCa) extending to the bladder, which aimed to evaluate the effects of CP on survival outcomes and improving quality of life (QoL) in these patients.

**Methods:**

A total of 27 PCa patients extending to the bladder were subjected to CP and followed up at regular intervals in our center. Prostate cancer-specific survival (PCSS) and prostate-specific antigen recurrence-free survival (PFS) were assessed by Kaplan–Meier analysis. Multivariate Cox regression was performed to evaluate clinical characteristics predicting survivals. QoL and pelvic symptoms were also evaluated.

**Results:**

Median PCSS was not reached over the period of follow-up. 5-year PCSS rate was 82.1%. Median PFS was 66.0 months. 5-year PFS rate was 58.5%. Multivariate analysis showed Gleason score (≥ 8) (hazard ratio (HR) 2.55, 95% confidence interval (CI) 1.28–4.04, *p* = 0.033), positive local lymph node status (HR 3.52, 95% CI 1.57–7.38, *p* = 0.006) and bladder muscle-invasion (HR 4.75, 95% CI 1.37–7.53, *p* < 0.001) were independent predictors of worse PCSS. The number of patients suffering pelvic symptoms was significantly decreased, and QoL scores were significantly down-regulated after surgeries.

**Conclusion:**

CP offered effective and durable palliation in patients of locally advanced prostate cancer with invasion of the bladder, providing better QoL and relieving local symptoms.

## Background

When prostate cancer invades the bladder, it can be regarded as clinical T4 stage and locally advanced prostate cancer (LAPCa) according to the latest classification system narrated in the European Association of Urology guidelines. To date, no studies have identified the most optimal treatment option in the absence of high-level evidence [1]. Generally, radical prostatectomy (RP) in selected patients with no tumor fixation to the pelvic wall or no invasion of the urethral sphincter is considered as part of multimodality therapies, including androgen deprivation therapy, new hormonal treatments, radiotherapy, chemotherapy and various combinations of these treatment modalities [2]. However, for those patients with tumor invading ureteric orifices and bladder outlet obstructions, such conservative treatments often result in a refractory state and patients may endure a lifelong dependence on ureteral stents, urethral catheters, or nephrostomy tubes [3, 4]. They have to endure multiple invasive procedures for routine tube exchange.

Scarce studies were reported on surgeries in treating LAPCa due to the traditional thought that the patients with LAPCa, especially those extending to the bladder would fare poorly. However, data from retrospective case series of LAPCa demonstrated over 60% cancer-specific survival (PCSS) at 15 years and over 75% overall survival (OS) at 10 years [5–7]. In addition, the substantially lower mortality in very high-risk LAPCa with exposure to RP suggested that radical treatment decreased mortality even for whom such treatment had been considered ineffective [8]. In an international multidisciplinary systematic review, when comparing RP with external beam radiotherapy, retrospective series reported benefits in OS and PCSS ranging from 10 to 28% and from 4 to 8%, respectively [1]. Therefore, RP was recommended as the primary treatment in high-risk and LAPCa as part of multimodal treatment.

We hypothesize that, for highly selected PCa patients with bladder invasion in the absence of fixing to the pelvic wall, or invading the urethral sphincter or rectum, or distant metastases, cystoprostatectomy (CP) may be a reasonable treatment option. At least, this will help them to get rid of local symptoms of bladder invasion and boring procedures of tube exchange during their whole lives. In 2005, a study by Leibovici et al. firstly reported the effectiveness of salvage CP for clinical T4 PCa patients with bladder invasion, providing palliation of lower urinary tract symptoms and 31 months of median PCSS [4]. Then in 2009, Kumazawa et al. also reported that CP might be a feasible treatment option to achieve excellent local control for patients with previously untreated PCa involving the bladder, even in the presence of pelvic lymph node metastasis [3]. Recently, two retrospective studies showed that CP was technically feasible in well-selected patients with acceptable morbidity and good short-term survival outcome, resulting in symptom relief in 90% of patients, covering 80% of their remaining lifetime [9, 10]. However, neither reports evaluated the effects of different presentations of clinical T4 PCa on survival, nor did they assess CP in improving quality of life (QoL) in patients. In the present study, the effects of CP on survival outcomes and improving QoL in these patients were evaluated.

## Methods

### Patients

The Ethics Committee of Shandong Provincial Hospital approved the study protocol. Informed consents were obtained from all participants before any traumatic procedure. Between January 2014 and June 2020, 28 PCa patients firstly diagnosed having bladder invasion without distant organ/lymph node metastasis, tumor fixation to the pelvic wall, or invasion of rectum/pelvic wall received CP in our center. The following exclusion criteria were applied to identify the inappropriate candidates for CP in the present study: the patients with distant visceral metastasis, distant lymph node metastasis, or tumor invasion of rectum and pelvic wall; life expectancy < 5 years; Eastern Cooperative Oncology Group performance status > 2; and the patients who received local therapy previously (external beam radiation therapy, brachytherapy, etc.).

All patients were diagnosed by preoperative transrectal needle biopsies. Bone scan, chest X-ray and pelvic magnetic resonance imaging (MRI) were used for preoperative staging. Bladder invasion was determined by MRI, cystoscopy and pathological analysis. All patients received preoperative therapies for three to six months, including neoadjuvant hormone therapy, i.e. complete androgen blockade (CAB) by using luteinizing hormone-releasing hormone analog and antiandrogen agents, and neoadjuvant chemotherapy, i.e. docetaxel. After surgeries, all patients were treated with androgen deprivation therapy, and part of the patients received chemotherapy or external beam radiation therapy.

### Surgical interventions

All operations were performed by two surgeons of our center. Both surgeons were skilled for many years in various types of CP, and well beyond learning curves for surgical techniques (> 300 CP procedures). Rectal neobladder (n = 10), ileal conduit (n = 11), and ureterocutaneostomies (n = 7) were performed. Rectal neobladder is a form of urine diversion from the lower urinary tract into the sigmoid colon. This continent rectal reservoir technique allows the storage and outflow of urine through the rectum, utilizing the anus for continence. The ureters should be located beneath the common iliac arteries and properly mobilized. Using an antirefluxing approach, both ureters are re-implanted into the tenia coli independently. For ileal conduit, T pouch is commonly employed in our center. It creates an antireflux approach with a smaller ileal segment, which eliminates the requirement for intussusception, preserves blood supply, and keeps urine away from the implanted ileal segment. Extended pelvic lymph nodes dissections, including the nodes within the obturator fossa located cranially and caudally to the obturator nerve, the nodes overlying the external iliac vessels, and the nodes medial and lateral to the internal iliac artery, were performed in all patients.

### Data collection

Basic pathophysiologic features of all patients, including age, prostate-specific antigen (PSA), Gleason score, results of bone scan, chest x-ray and pelvic MRI, pelvic symptoms (hematuria, obstructive voiding symptoms, pelvic pain, hydronephrosis and indwelling tubes), and QoL were recorded. After surgeries, the following parameters were recorded: Gleason score, PSA, lymph node status, seminal vesicle status, surgical margins, invasion depth of bladder wall, pelvic symptoms, complications and QoL. Perioperative complications were classified using the Clavien-Dindo system. All patients were followed up at three-monthly intervals in the first two years, then at six-monthly intervals, and assessed for PSA and pelvic symptoms. PCSS and PSA recurrence-free survival (PFS) were analyzed. PCSS was defined as the duration from the date of entering the study until death due to prostate cancer progression. PSA recurrence was defined by two consecutive PSA > 0.2 ng/mL and rising [11].

### Statistical analysis

Data were analyzed with SPSS 17.0 (IBM Software, USA). Continuous variables were expressed as mean ± standard error of mean and analyzed by the Student's t test. Patients were censored if they had not experienced the endpoint of interest by the last follow-up. Survival curves were estimated with the Kaplan–Meier method with log-rank test. Univariate analysis and multivariate Cox regression model were used to assess the influences of clinicopathological features of PCa and therapeutic strategies on PCSS and PFS. Chi-square test was applied to compare the occurrences of pelvic symptoms between preoperation and postoperation. Statistical significance was set at p < 0.05.

## Results

### Pathophysiologic characteristics of patients

One patient died of severe bleeding of the internal iliac artery caused by intraoperative lymph nodes dissection, which was excluded from the final analysis involving 27 cases left. The mean age of patients was 63.2 years. The mean serum PSA level was 40.2 ng/ml. Detailed perioperative parameters and pathophysiologic characteristics of patients are shown in Table 1. Follow-up time was 52.2 ± 18.3 months (median = 42.0).

**Table 1:** Clinical characteristics of patients

No. patients	27
Age (years)	63.2 ± 7.6
Operation time (hours)	4.4 ± 2.1
Blood loss (ml)	58.3 ± 36.9
Hospital stay (days)	12.2 ± 2.7
*Preoperative PSA*	
No. ≤ 20 ng/ml (%)	7 (25.9)
No. > 20 ng/ml (%)	20 (74.1)
*Pelvic symptoms*	
No. hematuria (%)	24 (88.9)
No. obstructive voiding symptoms (%)	22 (81.5)
No. pelvic pain (%)	19 (70.4)
No. Hydronephrosis (%)	23 (85.2)
No. Indwelling tubes (%)	25 (92.6)
*Therapeutic methods*	
No. Neo CAB + Post ADT (%)	5 (18.5)
No. Neo CAB + Post ADT + Post Che (%)	10 (37.0)
No. Neo CAB + Post ADT + Post Rad (%)	9 (33.3)
No. Neo Che + Post ADT + Post Che (%)	3 (11.2)
*Gleason score*^*a*^	
No. ≤ 6 (%)	2 (7.4)
No. 7 (%)	10 (37.0)
No. ≥ 8 (%)	15 (55.6)
*Local lymph node status*^*a*^	
No. pN0 (%)	9 (33.3)
No. pN1 (%)	18 (66.7)
*Seminal vesicle status*^*a*^	
No. negative (%)	10 (37.0)
No. positive (%)	17 (63.0)
*Surgical margins*^*a*^	
No. negative (%)	16 (59.3)
No. positive (%)	11 (40.7)
*Invasion depth of bladder wall*^*a*^	
No. non-muscle-invasion (%)	11 (40.7)
No. muscle-invasion (%)	16 (59.3)

^a^These issues were determined by postoperative pathological analyses. ADT, androgen deprivation therapy; Che, chemotherapy; PSA, prostate-specific antigen; CAB, maximal androgen blockade; Neo, neoadjuvant; Post, postoperative; Rad, radiation therapy

### Survival analysis

**Fig.1:**
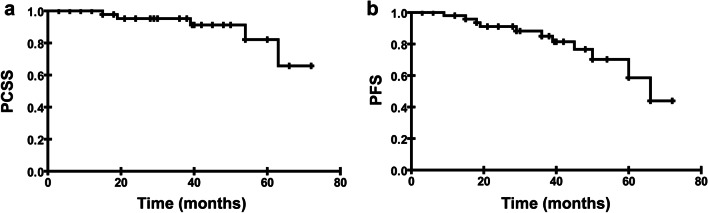
Survival analyses were assessed by Kaplan–Meier analysis. **a** Median PCSS was not reached over the period of follow-up. PCSS at 5-year was 82.1%. **b** Median PFS was 66.0 months. 5-year PFS rate was 58.5%

Five patients (18.5%) died as a direct result of tumor progression. Median PCSS was not reached over the period of follow-up. PCSS at 5-year was 82.1% (Fig. 1a). PSA recurrence after surgery was observed in 11 patients (40.7%). Median PFS was 66.0 months. 5-year PFS rate was 58.5% (Fig. 1b).

To determine the potential clinical features affecting prognosis, analyses of PCSS and PFS were stratified by preoperative PSA level, therapeutic method, Gleason score, local lymph node status, seminal vesicle status, surgical margin status and invasion depth of bladder wall, based upon postoperative pathological results.

Univariate analysis in Table 2 showed those patients with pN0 (*p* = 0.036) and non bladder wall muscle-invasion (*p* = 0.021) had significantly higher PCSS compared with patients with pN1 and muscle-invasion, respectively. PCSS was comparable in those patients stratified by preoperative PSA level (*p* = 0.504), by therapeutic method (*p* = 0.391), by Gleason score (*p* = 0.066), by seminal vesicle status (*p* = 0.406) and by surgical margin status (*p* = 0.338). Those patients with PSA ≤ 20 ng/ml (*p* = 0.031), smaller Gleason score (*p* = 0.015), negative surgical margins (*p* = 0.014) and non muscle-invasion (*p* = 0.038) presented significantly prolonged PFS compared with respective subgroups. While patients stratified by therapeutic method (*p* = 0.352), by local lymph node status (*p* = 0.219) and by seminal vesicle status (*p* = 0.266) had comparable PFS, respectively.

**Table 2:** Univariate survival analysis in patients

	PCSS	PFS		
	Median PCSS in months (95% CI)	*P* value	Median PFS in months (95% CI)	*P* value
*Preoperative PSA (ng/ml)*		0.504		0.031
≤ 20	67.2 (55.5–72.1)		67.8 (60.7–73.2)	
> 20	64.4 (58.6–71.0)		59.9 (55.3–69.6)	
*Gleason score*^a^		0.066		0.015
≤ 6	Not reached		Not reached	
7	67.8 (62.6–68.4)		63.7 (59.3–66.6)	
≥ 8	64.1 (57.1–65.6)		50.0 (39.4–57.8)	
*Therapeutic methods*		0.391		0.352
Neo CAB + Post ADT	Not reached		62.5 (57.2–70.1)	
Neo CAB + Post ADT + Post Che	71.3 (68.8–73.2)		64.3 (53.0–67.4)	
Neo CAB + Post ADT + Post Rad	66.9 (64.3–70.1)		61.7 (58.2–65.7)	
Neo Che + Post ADT + Post Che	67.9 (63.5–69.5)		65.0 (63.8–72.5)	
*Local lymph node status*^a^		0.036		0.219
pN0	72.2 (68.8–74.5)		68.4 (62.2–69.9)	
pN1	64.3 (61.1–67.0)		69.4 (65.7–72.6)	
*Seminal vesicle status*^a^		0.406		0.266
Negative	71.3 (67.7–72.7)		70.3 (66.2–72.3)	
Positive	68.6 (65.6–71.0)		69.8 (64.0–71.7)	
*Surgical margins*^a^		0.338		0.014
Negative	70.6 (66.7–71.9)		70.2 (65.4–72.8)	
Positive	68.8 (67.2–69.6)		59.7 (57.8–66.3)	
*Invasion depth of bladder wall*^a^		0.021		0.038
Non-muscle-invasion	68.8 (65.9–73.0)		Not reached	
Muscle-invasion	61.5 (54.2–68.1)		60.4 (55.6–67.1)	

^a^These issues were determined by postoperative pathological analyses. ADT, androgen deprivation therapy; Che, chemotherapy; CI, confidence interval; HR, hazard ratio; CAB, maximal androgen blockade; Neo, neoadjuvant; PCSS, prostate cancer-specific survival; PFS, PSA recurrence-free survival; Post, postoperative; PSA, prostate-specific antigen; Rad, radiation therapy

In multivariate Cox proportional hazards regression analysis, Gleason score (≥ 8) (*p* = 0.033), local lymph node status (pN1) (*p* = 0.006) and invasion depth of bladder wall (muscle-invasion) (*p* < 0.001) were significant predictors of worse PCSS. While Gleason score (= 7, *p* = 0.029; ≥ 8, *p* = 0.026), therapeutic methods (neoadjuvant chemotherapy + postoperative CAB + postoperative chemotherapy) (*p* = 0.041), local lymph node status (pN1) (*p* = 0.011) and invasion depth of bladder wall (muscle-invasion) (*p* = 0.020) were significant predictors of worse PFS. The multivariate analysis is detailed in Table 3.

**Table 3:** Multivariate survival analysis in patients

	PCSS		PFS	
	HR (95% CI)	*P* value	HR (95% CI)	*P* value
*Preoperative PSA (ng/ml)*				
≤ 20	Reference		Reference	
> 20	1.85 (0.76–3.88)	0.163	2.08 (0.88–3.05)	0.219
*Gleason score*^*a*^				
≤ 6	Reference		Reference	
7	1.15 (0.74–2.16)	0.061	2.28 (1.19–3.96)	0.029
≥ 8	2.55 (1.28–4.04)	0.033	1.96 (1.33–3.89)	0.026
*Therapeutic methods*				
Neo CAB + Post ADT	Reference		Reference	
Neo CAB + Post ADT + Post Che	1.57 (0.63–2.63)	0.673	0.96 (0.90–1.38)	0.363
Neo CAB + Post ADT + Post Rad	2.89 (0.55–5.01)	0.419	1.42 (0.79–1.78)	0.186
Neo Che + Post ADT + Post Che	1.80 (0.79–2.61)	0.309	1.17 (1.02–2.71)	0.041
*Local lymph node status*^*a*^				
pN0	Reference		Reference	
pN1	3.52 (1.57–7.38)	0.006	2.60 (1.47–3.38)	0.011
*Seminal vesicle status*^*a*^				
Negative	Reference		Reference	
Positive	2.16 (0.76–5.04)	0.266	0.89 (0.61–1.58)	0.068
*Surgical margins*^*a*^				
Negative	Reference		Reference	
Positive	1.26 (0.58–2.51)	0.075	2.24 (0.58–3.06)	0.421
*Invasion depth of bladder wall*^*a*^				
Non-muscle-invasion	Reference		Reference	
Muscle-invasion	4.75 (1.37–7.53)	< 0.001	1.46 (1.06–3.02)	0.020

^a^These issues were determined by postoperative pathological analyses. ADT**,** androgen deprivation therapy; Che, chemotherapy; CI, confidence interval; HR, hazard ratio; CAB, maximal androgen blockade; Neo, neoadjuvant; PCSS, prostate cancer-specific survival; PFS, PSA recurrence-free survival; Post, postoperative; PSA, prostate-specific antigen; Rad, radiation therapy

### Effects of CP on pelvic symptoms and QoL

The numbers of patients with enduring major pelvic symptoms, including hematuria, obstructive voiding symptoms, pelvic pain, hydronephrosis and the number of patients with the indwelling of tubes were significantly less after surgeries (Table 4).

**Table 4:** Effects of CP on occurrences of pelvic symptoms

	Preoperation	Postoperation	*P* value
No. Hematuria (%)	24 (88.9)	4 (14.8)	< 0.001
No. Obstructive voiding symptoms (%)	22 (81.5)	2 (7.4)	< 0.001
No. Pelvic pain (%)	19 (70.4)	7 (25.9)	< 0.001
No. Hydronephrosis (%)	23 (85.2)	11 (40.7)	0.018
No. Patients of indwelling tubes (%)	25 (92.6)	8 (29.6)	< 0.001

*CP* Cystoprostatectomy

For QoL evaluation, CP significantly down-regulated scores, from 5.5 ± 0.4 at baseline to 2.1 ± 0.3 (*p* < 0.001), 1.8 ± 0.6 (*p* < 0.001), 1.9 ± 0.3 (*p* < 0.001), 1.9 ± 0.5 (*p* < 0.001), 2.0 ± 0.6 (*p* < 0.001), and 1.8 ± 0.3 (*p* < 0.001) at 6, 12, 24, 36, 48, 60 months after surgeries.

### Complications

As mentioned above, one patient died of severe intraoperative bleeding. Two patients experienced Clavien grade IIIb (rectal injury, n = 2, 7.4%) that required surgical repair intraoperatively. During the postoperative follow-up, Clavien grades II and IIIa complications developed in 25.9% (wound infection, n = 3, 11.1%; urinary tract infection, n = 3, 11.1%; acute pyelonephritis, n = 1, 3.7%) and 33.3% (prolonged intestinal paralysis, n = 5, 18.5%; anastomotic stricture, n = 2, 7.4%; enterocutaneous fistula, n = 2, 7.4%) of patients, respectively.

## Discussion

When performing CP for PCa involving bladder, some medical centers might claim the surgical significance or the possibility of overtreatment. However, the refractory and disappointing state after traditional therapies often reduces the patients’ QoL seriously by recurrent lower urinary tract symptoms and enduring a lifelong dependence on tubes and catheters. Improving local control and providing a better QoL to the patients are imperative clinical goals independent of the survival outcome. Up to date, CP as a treatment strategy for clinical T4 PCa invading into bladder, is still controversial without full evaluation. Only limited number of literatures have been published [3, 4], partly because of a decreased incidence of T4 disease, but mainly due to stubborn thoughts of poor prognosis of these patients. However, data from LAPCa cohort studies showed a 15-year PCSS of 60% and a 10-year OS of 75% [5–7]. The oncological effectiveness of RP as part of a multi-modal treatment strategy for LAPCa remains unknown. A prospective phase III randomized controlled trial comparing RP against primary external beam radiotherapy and androgen deprivation therapy among patients with LAPCa is currently recruiting (https://clinicaltrials.gov/ct2/show/NCT02102477). Several retrospective studies showed that selected T4 patients who received RP acquired better OS and PCSS than those undergoing no surgery or radiation therapy [12, 13]. In 2015, CP was performed to treat castration-resistant prostate cancer with infiltration of dorsal bladder by Axel Heidenreich and his colleagues [14]. The mean OS in their patient cohort was 20.4 (1–28) months and the mean symptom-free survival was 15.3 (1–25) months, covering 75% of the total survival time. The authors concluded that palliative radical CP was a challenging but feasible local treatment option in well-selected bladder invasive castration-resistant prostate cancer patients if performed by experienced hands. In our follow-up with a median period of 42.0 months, the 5-year PCSS rate of patients received CP reached 82.1%, comparable with 87.1% in the previous study [3]. Omar Fahmy et al. reported that 5-year PCSS rates of patients with LAPCa who were treated with RP, radiation therapy and hormone therapy were 94.2%, 95.7%, and 78.5%, respectively [15]. Probably, more advanced and higher risk of tumors in the participated patients in the present study resulted in discrepancies. The significantly lower QoL scores after surgeries suggested that CP had a role in improving the quality of life in patients with PCa extending to the bladder. Moreover, our results supported the role of CP in relieving pelvic symptoms, especially in ceasing dependence on tubes, which is consistent with previous reports [3, 4].

CP itself is still a technically challenging procedure. In a previous study of salvage CP for radiation failure in PCa, one early death (12.5%) occurred from a pulmonary embolism within 60 days of surgery, accompanied by 4 (50%) of rectal injury [16]. In the present study, one immediate death occurred due to a massive hemorrhage of the internal iliac artery during lymph node dissection. Severe adhesion of lymph nodes to internal iliac vessels led to this serious consequence, which was not unique in CP for PCa extending to the bladder. Many studies have shown that the more extensive the lymph node dissection, the greater the adverse outcomes in terms of blood loss [17]. Only 7.4% of patients suffered postoperative rectal injury in the present study because patients with tumor invasion of rectum were excluded from the series.

Our study showed that the entered patients had frequent lymph node metastasis (66.7%). Although pN1 was a predictor of worse prognosis as shown in the present study, the median PCSS even with lymph node metastasis was 64.3 months. One of the limitations of our study was lacking a controlling trial of patients subjected to non-CP interventions, making it impossible to compare the effects of CP on PCa patients with conservative therapies. Jutta Engel et al. [18] reported that RP was a strong independent predictor of survival in patients with node-positive PCa, improving OS by 24% versus those patients with aborted RP. There have been several retrospective observational studies showing dramatic improvements in PCSS in favor of RP versus non-RP in patients who were found to be lymph node metastasis [19]. From the results of these studies and our study, CP is also suggested to be applicable in T4 PCa patients with node-positive disease. Moreover, Fizazi K et al. reported upfront usage of docetaxel only improved clinical relapse-free survival for T4 patients, with no long-term survival benefit [20]. This conclusion was further confirmed in the present study. Multivariate analysis demonstrated that PCSS was not improved by adding neoadjuvant chemotherapy versus neoadjuvant CAB. In addition, choices of combined postoperative adjuvant therapies did not affect PCSS.

Patients entered in this study had 55.6% of high-grade tumors (Gleason score ≥ 8), which are commonly considered as potentially significant risk factors for poor outcomes. Nevertheless, some retrospective case series reported good outcomes after RP for patients with high-grade PCa in combination with radiation plus hormonal therapy [21, 22]. In the present study, the median PCSS with Gleason score ≥ 8 was 64.1 months. However, for the patients who received CP, Gleason score ≥ 8 was a predictor of worse PCSS and PFS.

Muscle-invasive bladder cancer is a frequently occurring disease with a high mortality rate despite optimal treatment due to the common involvement of nodes and high-grade urothelial carcinomas [23, 24]. For the first time in the world, we assessed CP on the prognosis of PCa regarding invasion depth of the bladder wall. For the patients received CP, the median PCSS of those with bladder muscle-invasion was 61.5 months, shorter than those with non-muscle-invasion (68.8 months). Multivariate analysis suggested bladder muscle-invasion was an independent predictor of poor outcomes for patients who received CP.

Several limitations should be stated before the conclusion. The main limitation is the small number of entered patients because of the low incidence of T4 PCa nowadays. Nevertheless, the involved patients in the present study were still more than in the previous report, 17 in Kumazawa's study [3]. Moreover, as narrated above, our study was lacking a controlling trial of patients received traditional therapies due to the limited number of participants. Hence, comparisons of CP and conservative therapies on PCa patients were not performed.

## Conclusion

Our results supported the concept that CP offered effective and durable palliation in patients of LAPCa with invasion of bladder, providing better QoL and relieving local symptoms. With the summary of the total cases, Gleason score ≥ 8, local lymph node status of N1, and muscle-invasion of bladder wall were independent predictors of worse prognosis in these patients subjected to CP.

## Data Availability

The datasets used and/or analysed during the current study available from the corresponding author on reasonable request.
